# Predictors of unfavorable 3-month functional outcome following intravenous thrombolysis with alteplase in anterior circulation acute ischemic stroke: a prospective cohort study

**DOI:** 10.3389/fnins.2026.1818367

**Published:** 2026-07-08

**Authors:** Chaowei Wang, Shuo Wang, Yunqing Mao, Li Liu, Guang Yao

**Affiliations:** 1 Department of Neurology, The First Affiliated Hospital of Henan Medical University, Weihui, Henan, China; 2 Henan Key Laboratory of Neural Repair and Protein Modification, Henan International Joint Laboratory for Neural Repair of Alzheimer’s Disease, Henan Engineering Research Center for Neural Repair, Henan Engineering and Technology Research Center for Neural Repair, Henan Key Medical Laboratory of Neurology, Xinxiang, China; 3 Department of Neuroelectrophysiology, First Affiliated Hospital of Henan Medical University, Weihui, Henan, China; 4 Department of Mathematics and Statistics, Xinxiang University, Xinxiang, Henan, China

**Keywords:** acute ischemic stroke, alteplase, clot burden score, collateral circulation, C-reactive protein, d-dimer, functional outcome, intravenous thrombolysis

## Abstract

**Background:**

Intravenous thrombolysis with alteplase remains the standard reperfusion strategy for acute ischemic stroke (AIS), yet many patients still experience unfavorable outcomes despite timely treatment, underscoring the need for reliable multimodal prognostic markers.

**Objective:**

To identify independent clinical, laboratory, and neuroimaging predictors of unfavorable 3-month functional outcome in anterior circulation AIS treated with alteplase, and to develop, internally validate, and benchmark an integrated multivariable model in accordance with TRIPOD.

**Methods:**

This prospective single-center cohort study enrolled 268 consecutive patients with anterior circulation AIS receiving intravenous alteplase within 4.5 h of symptom onset (March 2022–February 2025). Three-month outcome was assessed by the modified Rankin Scale (mRS 0–2 favorable; 3–6 unfavorable) using validated structured instruments administered by blinded raters. Multivariable logistic regression was performed, and the final model was internally validated by 1,000-replicate bootstrap with optimism correction and shrinkage, evaluated by decision curve analysis (DCA), rendered into a nomogram, and benchmarked head-to-head against three previously published reference models using the DeLong test.

**Results:**

Of 268 patients, 99 (36.9%) experienced unfavorable outcomes. Seven independent predictors were identified: early neurological deterioration (adjusted OR 3.45, 95% CI 2.01–5.92), large infarction exceeding one-third of the middle cerebral artery territory (OR 2.78, 1.58–4.89), baseline NIHSS (OR 1.14 per point, 1.07–1.22), poor Tan collateral score (OR 2.31, 1.34–3.98), low clot burden score (OR 2.15, 1.28–3.61), elevated D-dimer (OR 2.19, 1.29–3.72), and elevated CRP (OR 1.87, 1.11–3.15). The model achieved an apparent AUC of 0.847 (95% CI 0.801–0.893) and an optimism-corrected AUC of 0.831 (0.785–0.877) on bootstrap validation, with satisfactory calibration (Hosmer–Lemeshow *P* = 0.394). DCA showed positive net benefit across threshold probabilities of 0.15–0.75, and the model exceeded the recalibrated Hu, Ping, and Lv models.

**Conclusion:**

A multimodal panel integrating clinical, inflammatory, coagulation, and neuroimaging parameters independently predicts unfavorable 3-month outcome following intravenous thrombolysis in anterior circulation AIS. The findings are hypothesis-generating pending external validation in independent multicenter cohorts.

## 1 Introduction

Acute ischemic stroke (AIS) constitutes a leading cause of long-term disability and mortality worldwide, imposing a formidable burden on healthcare systems and patients alike (Feigin et al., 2022; GBD 2019 Stroke Collaborators, 2021). The administration of intravenous recombinant tissue plasminogen activator (alteplase) within the established 4.5-h therapeutic window remains the only pharmacological reperfusion strategy endorsed by international guidelines for the treatment of eligible AIS patients (Berge et al., 2021; Powers et al., 2019). Despite the proven efficacy of intravenous thrombolysis in dissolving occluding thrombi and restoring cerebral perfusion, a considerable proportion of treated patients ranging from 30 to 50% in published registries fail to achieve a favorable functional outcome, underscoring the heterogeneous nature of treatment response and the multifactorial determinants of post-stroke recovery (Fekete et al., 2023; Zhai et al., 2023).

The clinical trajectory following intravenous thrombolysis is governed by a complex interplay of patient-specific factors, including baseline neurological severity, vascular anatomy, thrombus characteristics, systemic inflammatory responses, and coagulation dynamics (Emberson et al., 2014; Xiong et al., 2021). In recent years, accumulating evidence has elucidated the prognostic relevance of several biomarkers and imaging parameters in this context. Elevated D-dimer concentrations, a surrogate marker of ongoing thrombotic activity and fibrinolytic resistance, have been increasingly recognized as predictors of poor functional outcome and symptomatic intracranial hemorrhage following intravenous thrombolysis (Jin et al., 2022; Li et al., 2022). Similarly, C-reactive protein (CRP), a nonspecific acute-phase reactant reflective of systemic inflammation, has been associated with larger infarct volumes, early neurological deterioration, and diminished prospects of functional recovery in AIS patients receiving reperfusion therapy (VanGilder et al., 2014; Welsh et al., 2008).

From a neuroimaging perspective, advances in computed tomography angiography (CTA) have enabled the quantitative assessment of intracranial thrombus burden and collateral blood flow, both of which exert a decisive influence on the extent of salvageable penumbral tissue and, consequently, on clinical outcome (Menon et al., 2011; Puetz et al., 2008). The clot burden score (CBS), a semi-quantitative CTA-based metric originally developed to quantify intracranial thrombus extent in the anterior circulation, has demonstrated robust predictive value for both early neurological improvement and long-term functional outcome after thrombolysis and thrombectomy (Szegedi et al., 2021). Likewise, the Tan collateral score, which grades the pial arterial filling distal to an occluding thrombus, has emerged as a powerful determinant of tissue viability and functional prognosis, with poor collateral status independently associated with larger completed infarctions and higher rates of unfavorable outcome (Maas et al., 2009; Tan et al., 2009).

Several prognostic frameworks for post-thrombolysis outcomes have been proposed over the past decade, including the laboratory-weighted nomogram of Hu et al. (2022), the inflammation-based composite of Ping et al. (2022), and the clinical-radiological score of Lv et al. (2020), each of which has advanced the prognostic literature in important ways. Despite the growing body of literature investigating individual predictors, relatively few prospective studies have simultaneously evaluated the combined prognostic contribution of clinical, laboratory, and imaging parameters in a unified multimodal framework specifically within the context of intravenous thrombolysis, and fewer still have benchmarked their performance head-to-head against established reference models within the same dataset. Furthermore, earlier investigations have been limited by retrospective designs, modest sample sizes, or the exclusion of CTA-derived vascular metrics from the analytical models (Lin et al., 2018; Siedler et al., 2019). Accordingly, the present study was designed to prospectively identify independent predictors of unfavorable 3-month functional outcome in a consecutive cohort of anterior circulation AIS patients treated with intravenous alteplase, integrating clinical, biochemical, and advanced neuroimaging variables within a comprehensive multivariable logistic regression model developed and reported in accordance with the TRIPOD statement (Collins et al., 2015) and benchmarked against three previously published prediction models. We hypothesized that a composite panel comprising markers of neurological severity, inflammation, coagulation activation, and vascular status would yield superior prognostic discrimination compared with models relying on clinical parameters alone.

## 2 Materials and methods

### 2.1 Study design and ethical approval

This prospective, single-center, observational cohort study was conducted at the Department of Neurology, the First Affiliated Hospital of Henan Medical University, Weihui City, Henan Province, China, between March 2022 and February 2025. The study protocol was approved by the Ethics Committee of the First Affiliated Hospital of Henan Medical University (approval reference EC-LW26-107; updated certificate issued on 7 May 2026) and was conducted in accordance with the principles of the Declaration of Helsinki, with the manuscript prepared in accordance with the TRIPOD statement for the transparent reporting of multivariable prediction models (Collins et al., 2015). Written informed consent was obtained from all patients or their legally authorized representatives prior to enrollment.

### 2.2 Study population

Consecutive patients presenting with anterior circulation AIS who were eligible for and treated with intravenous alteplase were screened for inclusion. The inclusion criteria were as follows: (i) age ≥ 18 years; (ii) clinical diagnosis of anterior circulation AIS confirmed by neuroimaging; (iii) administration of intravenous alteplase (0.9 mg/kg, maximum 90 mg) within 4.5 h of symptom onset in accordance with prevailing international guidelines; (iv) baseline non-contrast CT and CTA performed prior to thrombolysis; and (v) availability of 3-month functional outcome data. Exclusion criteria encompassed patients with posterior circulation strokes, those who received concomitant endovascular thrombectomy, patients treated with tenecteplase rather than alteplase, individuals with pre-existing functional disability (pre-stroke mRS ≥ 2), patients lost to follow-up, and those with incomplete baseline laboratory or imaging data. After applying these criteria, 268 patients were included in the final analysis.

The decision to exclude patients who underwent concomitant endovascular thrombectomy was deliberate and methodologically motivated, since our primary objective was to delineate prognostic determinants in the population managed with intravenous alteplase as the sole reperfusion strategy and thereby avoid the confounding influence of mechanical recanalization on functional trajectories. In our institution during the enrollment period, endovascular therapy was not pursued in patients with proximal occlusion presenting within the 4.5-h window for one of several documented reasons, namely the absence of a confirmed large-vessel occlusion satisfying institutional thrombectomy criteria on initial CTA, formal contraindications to endovascular intervention, declination of the procedure by the patient or their family, or logistical constraints related to inter-hospital transfer to the regional comprehensive stroke center. The implications of this design choice for the generalizability of the findings to contemporary large-vessel occlusion populations are explicitly addressed among the limitations.

### 2.3 Data collection and clinical assessment

At the time of hospital admission, a standardized data collection form was used to document baseline demographics (age, sex, body mass index), vascular risk factors (hypertension, diabetes mellitus, dyslipidemia, atrial fibrillation, coronary artery disease, previous stroke or transient ischemic attack, smoking status, and alcohol consumption), and clinical parameters including systolic and diastolic blood pressure, heart rate, blood glucose levels, and onset-to-needle time. Neurological severity was assessed at admission using the National Institutes of Health Stroke Scale (NIHSS) by a certified stroke neurologist. Stroke etiology was classified according to the Trial of Org 10,172 in Acute Stroke Treatment (TOAST) criteria. Early neurological deterioration (END) was defined as an increase in NIHSS score of ≥ 4 points within the first 24 h following thrombolysis.

### 2.4 Laboratory investigations

Venous blood samples were obtained at the time of admission, prior to alteplase administration. Standard laboratory analyses included complete blood count (white blood cell count, hemoglobin, platelet count), coagulation profile (D-dimer, fibrinogen, international normalized ratio), inflammatory markers (C-reactive protein), metabolic parameters (blood glucose, glycated hemoglobin, serum creatinine, lipid panel including total cholesterol, low-density lipoprotein cholesterol, and triglycerides), and homocysteine levels. D-dimer was quantified using an immunoturbidimetric assay (reference range < 0.5 mg/L), and high-sensitivity CRP was measured by latex-enhanced nephelometry (reference range < 5 mg/L).

### 2.5 Neuroimaging assessment

All patients underwent non-contrast CT of the brain followed by CTA of the intracranial and extracranial vasculature prior to thrombolysis. Imaging was reviewed independently by two experienced neuroradiologists who were blinded to the clinical outcomes. The following imaging parameters were systematically assessed: (i) the Alberta Stroke Program Early CT Score (ASPECTS) on non-contrast CT; (ii) the presence of large infarction, defined as hypodensity involving more than one-third of the middle cerebral artery (MCA) territory and corresponding to an ASPECTS value of ≤ 7 on baseline non-contrast CT, in keeping with established radiographic conventions for early ischemic change; (iii) the clot burden score (CBS), a 10-point CTA-based score quantifying the extent of intracranial thrombus in the anterior circulation, with lower scores indicating greater thrombus burden; (iv) the Tan collateral score, grading pial arterial filling on CTA as absent (grade 0), filling ≤ 50% of the MCA territory (grade 1), filling > 50% but < 100% (grade 2), or complete filling (grade 3), with grades 0–1 classified as poor collateral circulation and grades 2–3 as good; and (v) the presence and site of proximal large vessel occlusion (internal carotid artery or M1 segment). Post-thrombolysis follow-up imaging was obtained at 24 ± 6 h to assess for hemorrhagic transformation, classified according to the Heidelberg Bleeding Classification. Symptomatic intracranial hemorrhage (sICH) was defined as any parenchymal hemorrhage associated with an increase in NIHSS score of ≥ 4 points. CT perfusion imaging and angiographic recanalization assessment were not routinely performed within our institutional acute stroke workflow during the enrollment period, since baseline CTA constituted the standard advanced vascular imaging modality, and consequently quantitative ischemic core volume, final infarct volume, and angiographic recanalization status could not be reliably ascertained for the present cohort.

### 2.6 Outcome assessment

The primary outcome measure was functional status at 3 months following stroke onset, evaluated using the mRS by trained stroke nurses or research neurologists who were blinded to baseline clinical and imaging data. In-person evaluations were conducted using the simplified modified Rankin Scale questionnaire (smRSq) of Bruno and colleagues (Bruno et al., 2011), a validated structured instrument with demonstrated robust inter-rater reliability and concordance with the conventional mRS. When in-person assessment was not feasible, the standardized Rankin Focused Assessment–Ambulation (RFA-A) telephone interview (Saver et al., 2010) was administered; this validated structured instrument has been shown to yield mRS estimates concordant with those derived from face-to-face evaluation. To minimize inter-rater variability, all assessors completed certified mRS training prior to study commencement, and a random 10% sample of cases underwent independent re-scoring by a second blinded assessor, yielding a weighted kappa statistic of 0.86, indicating excellent agreement. A favorable functional outcome was defined as mRS 0–2 (functional independence), and an unfavorable functional outcome was defined as mRS 3–6 (moderate-to-severe disability or death).

### 2.7 Statistical analysis

Statistical analyses were conducted in accordance with the Academic Emergency Medicine consensus guidelines for the reporting of observational studies. Continuous variables were expressed as mean ± standard deviation or median with interquartile range (IQR) as appropriate based on the Shapiro–Wilk test of normality. Categorical variables were expressed as frequencies and percentages. Univariate comparisons between the favorable and unfavorable outcome groups were performed using the independent samples *t*-test or Mann–Whitney U test for continuous variables and the chi-square test or Fisher’s exact test for categorical variables.

All variables demonstrating a *P*-value < 0.10 in univariate analysis were entered into a multivariable binary logistic regression model employing a backward stepwise elimination procedure (removal criterion: *P* > 0.10) to identify independent predictors of unfavorable 3-month outcome. The results were expressed as adjusted odds ratios (ORs) with 95% confidence intervals (CIs). The discriminative performance of the final multivariable model was evaluated by the area under the receiver operating characteristic (ROC) curve (AUC), and model calibration was assessed using the Hosmer–Lemeshow goodness-of-fit test, with *P* > 0.05 indicating acceptable calibration. Collinearity among predictor variables was evaluated using variance inflation factors (VIF), with VIF < 5 considered acceptable. Given that the proportion of missingness for any single variable was < 3%, missing data were managed using a complete-case analytical approach. Little’s missing-completely-at-random (MCAR) test did not provide evidence against an MCAR pattern (χ^2^ = 18.62, *P* = 0.241), and the robustness of the complete-case estimates was confirmed by a sensitivity analysis based on multiple imputation by chained equations (*m* = 20 imputations). Internal validation of the final model was undertaken by 1,000-replicate bootstrap resampling to derive optimism-corrected estimates of discrimination and calibration, with linear shrinkage of the regression coefficients applied to mitigate residual optimism. Decision curve analysis (DCA) (Vickers and Elkin, 2006) was performed to evaluate the net clinical benefit of the model across a clinically pertinent range of threshold probabilities, and a nomogram was constructed from the final model coefficients to facilitate bedside risk estimation. Discriminative performance was further benchmarked against three previously published prediction models Hu et al. (2022), Ping et al. (2022), and Lv et al. (2020), each of which was recalibrated within the present cohort, and pairwise differences in AUC were assessed using the DeLong test for paired ROC curves (DeLong et al., 1988). Pre-specified subgroup analyses with tests for interaction were performed across clinically relevant strata, including age, sex, NIHSS severity, onset-to-needle time, hypertension, diabetes mellitus, atrial fibrillation, and collateral status. All statistical tests were two-sided, and a *P*-value < 0.05 was considered statistically significant. Analyses were performed using SPSS version 26.0 (IBM Corporation, Armonk, NY) and R version 4.2.1 (R Foundation for Statistical Computing, Vienna, Austria), with the rms, pROC, and rmda packages employed for internal validation, ROC comparisons, and decision curve analysis.

## 3 Results

### 3.1 Baseline characteristics of the study population

**FIGURE 1:**
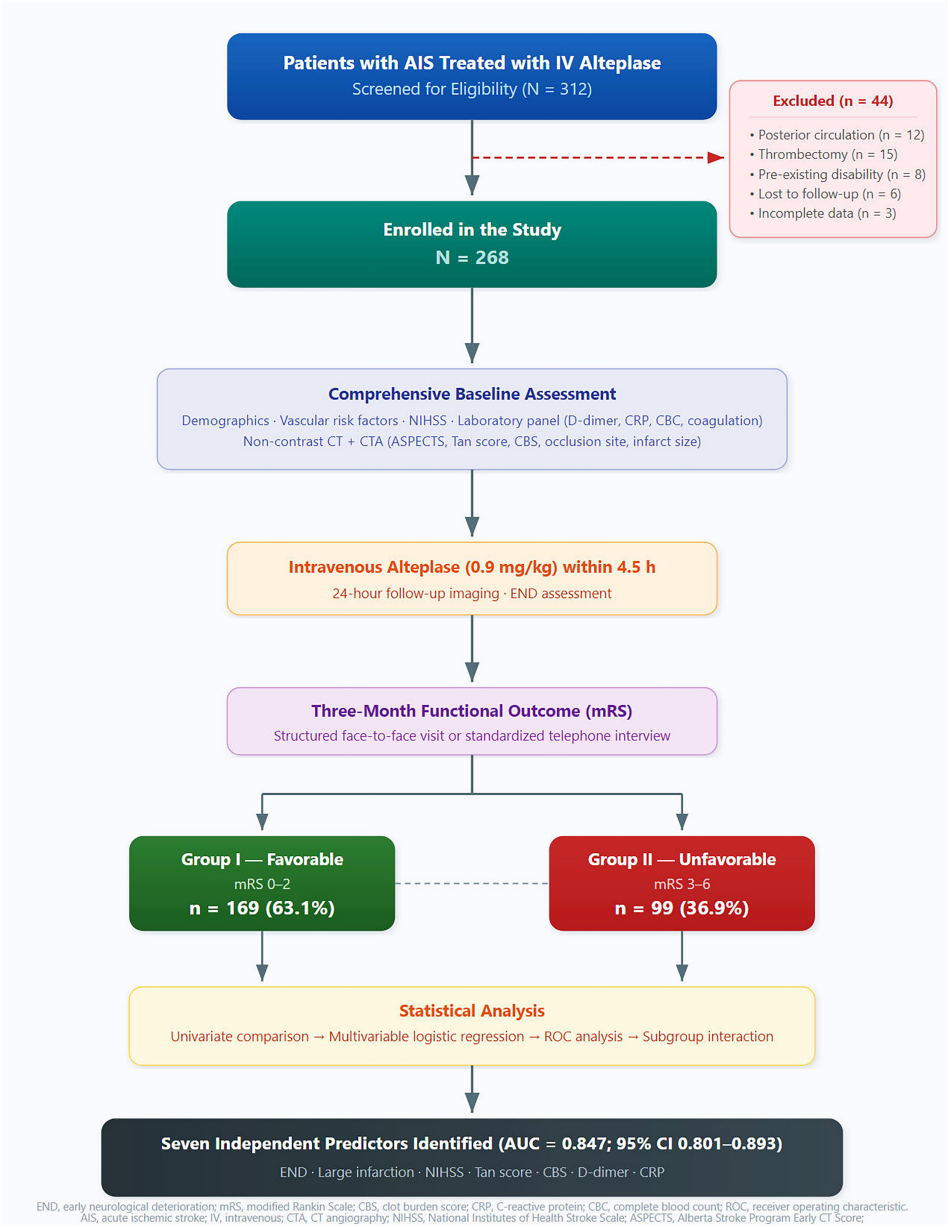
Study flowchart depicting patient enrollment, exclusion criteria, and group allocation. A total of 312 patients with acute ischemic stroke treated with intravenous alteplase were screened. After applying exclusion criteria, 268 patients were included and stratified into the favorable outcome group (mRS 0–2; *n* = 169, 63.1%) and the unfavorable outcome group (mRS 3–6; *n* = 99, 36.9%) based on 3-month modified Rankin Scale assessment. AIS, acute ischemic stroke; IV, intravenous; CTA, computed tomography angiography; mRS, modified Rankin Scale; END, early neurological deterioration; CBS, clot burden score; CRP, C-reactive protein.

A total of 312 patients with anterior circulation AIS who received intravenous alteplase during the study period were screened, of whom 268 met the inclusion criteria and were included in the final analysis (Figure 1). Forty-four patients were excluded owing to posterior circulation strokes (*n* = 12), concomitant endovascular thrombectomy (*n* = 15), pre-existing disability (*n* = 8), loss to follow-up (*n* = 6), and incomplete data (*n* = 3). At 3-month follow-up, 169 patients (63.1%) achieved favorable functional outcomes (mRS 0–2) and 99 patients (36.9%) experienced unfavorable outcomes (mRS 3–6). The distribution of individual mRS scores is depicted in Supplementary Figure 1.

The mean age of the entire cohort was 62.4 ± 12.8 years, with 156 patients (58.2%) being male. Patients in the unfavorable outcome group were significantly older and had a higher prevalence of hypertension, diabetes mellitus, and atrial fibrillation compared with those in the favorable outcome group (Table 1). No significant differences were observed between the two groups with regard to sex, dyslipidemia, coronary artery disease, prior stroke or transient ischemic attack, smoking status, or alcohol consumption (Supplementary Table 1).

**TABLE 1:** Baseline demographic, clinical, and risk factor characteristics of the study population.

Variable	Total (*n* = 268)	Favorable (*n* = 169)	Unfavorable (*n* = 99)	*P*-value
Demographics				
Age, years (mean ± SD)	62.4 ± 12.8	58.7 ± 11.9	68.7 ± 12.2	**< 0.001**
Male sex, n (%)	156 (58.2)	102 (60.4)	54 (54.5)	0.352
Vascular risk factors, n (%)				
Hypertension	178 (66.4)	104 (61.5)	74 (74.7)	**0.025**
Diabetes mellitus	89 (33.2)	48 (28.4)	41 (41.4)	**0.026**
Atrial fibrillation	72 (26.9)	38 (22.5)	34 (34.3)	**0.031**
Dyslipidemia	112 (41.8)	68 (40.2)	44 (44.4)	0.497
Clinical presentation				
NIHSS score, median (IQR)	9 (5–15)	6 (4–9)	14 (10–19)	**< 0.001**
Systolic BP, mmHg (mean ± SD)	152.3 ± 24.6	148.7 ± 22.8	158.4 ± 26.1	**0.002**
Blood glucose, mmoL/L, median (IQR)	6.8 (5.6–8.9)	6.3 (5.4–7.8)	7.8 (6.1–10.5)	**< 0.001**
Onset-to-needle time, min, median (IQR)	165 (120–210)	152 (110–195)	185 (140–235)	**0.001**
Early neurological deterioration, n (%)	78 (29.1)	28 (16.6)	50 (50.5)	**< 0.001**
Stroke etiology (TOAST), n (%)				
Large-artery atherosclerosis	98 (36.6)	58 (34.3)	40 (40.4)	0.314
Cardioembolism	82 (30.6)	46 (27.2)	36 (36.4)	0.109
Small-vessel occlusion	42 (15.7)	32 (18.9)	10 (10.1)	0.054
Other/undetermined	46 (17.2)	33 (19.5)	13 (13.1)	0.172

SD, standard deviation; IQR, interquartile range; NIHSS, National Institutes of Health Stroke Scale; BP, blood pressure; TOAST, Trial of Org 10,172 in Acute Stroke Treatment. Bold *P*-values indicate statistical significance (*P* < 0.05).

### 3.2 Clinical presentation and treatment parameters

The median baseline NIHSS score was significantly higher in the unfavorable outcome group than in the favorable outcome group [14 (IQR 10–19) vs. 6 (IQR 4–9); *P* < 0.001], reflecting a markedly greater neurological deficit at the time of presentation. Patients with unfavorable outcomes also exhibited higher systolic blood pressure (158.4 ± 26.1 vs. 148.7 ± 22.8 mmHg; *P* = 0.002), elevated admission blood glucose levels [7.8 (IQR 6.1–10.5) vs. 6.3 (IQR 5.4–7.8) mmoL/L; *P* < 0.001], and longer onset-to-needle times [185 (IQR 140–235) vs. 152 (IQR 110–195) min; *P* = 0.001]. Early neurological deterioration occurred in 78 patients (29.1%) overall and was profoundly more frequent in the unfavorable outcome group (50.5% vs. 16.6%; *P* < 0.001) (Table 1).

### 3.3 Laboratory findings

Comprehensive laboratory comparisons revealed that patients in the unfavorable outcome group had significantly elevated D-dimer concentrations [1.18 (IQR 0.68–2.05) vs. 0.42 (IQR 0.28–0.71) mg/L; *P* < 0.001] and CRP levels [12.5 (IQR 6.1–22.8) vs. 3.8 (IQR 1.9–7.2) mg/L; *P* < 0.001] compared with those who achieved favorable outcomes. Additionally, the unfavorable outcome group demonstrated higher white blood cell counts, fibrinogen levels, homocysteine concentrations, glycated hemoglobin values, and serum creatinine levels, whereas hemoglobin was modestly lower (Table 2 and Supplementary Table 2). The distributional differences in D-dimer, CRP, and NIHSS between the two outcome groups, selected as illustrative biomarkers spanning the coagulation, inflammatory, and clinical-severity domains, are visually depicted in Supplementary Figure 2.

**TABLE 2:** Key laboratory and neuroimaging parameters by outcome group.

Variable	Total (*n* = 268)	Favorable (*n* = 169)	Unfavorable (*n* = 99)	*P*-value
Laboratory parameters				
D-dimer, mg/L, median (IQR)	0.62 (0.32–1.28)	0.42 (0.28–0.71)	1.18 (0.68–2.05)	**< 0.001**
CRP, mg/L, median (IQR)	6.4 (2.5–14.8)	3.8 (1.9–7.2)	12.5 (6.1–22.8)	**< 0.001**
WBC count, × 10^9^/L (mean ± SD)	8.6 ± 3.2	7.8 ± 2.8	9.9 ± 3.5	**< 0.001**
Fibrinogen, g/L (mean ± SD)	3.42 ± 1.08	3.18 ± 0.92	3.83 ± 1.22	**< 0.001**
Homocysteine, μmoL/L, median (IQR)	14.2 (10.8–19.5)	12.8 (9.6–16.4)	17.5 (12.8–24.2)	**< 0.001**
HbA1c, % (mean ± SD)	6.4 ± 1.3	6.1 ± 1.1	6.9 ± 1.5	**< 0.001**
Neuroimaging parameters				
Large infarction ( > 1/3 MCA, ASPECTS ≤ 7), n (%)	86 (32.1)	32 (18.9)	54 (54.5)	**< 0.001**
ASPECTS, median (IQR)	8 (6–9)	9 (7–10)	6 (5–8)	**< 0.001**
Poor Tan score (grade 0–1), n (%)	80 (29.9)	30 (17.7)	50 (50.5)	**< 0.001**
CBS, median (IQR)	7 (5–9)	8 (6–9)	5 (3–7)	**< 0.001**
Proximal occlusion (ICA/M1), n (%)	108 (40.3)	52 (30.8)	56 (56.6)	**< 0.001**
Hemorrhagic transformation, n (%)	34 (12.7)	10 (5.9)	24 (24.2)	**< 0.001**
Symptomatic ICH (sICH), n (%)	12 (4.5)	2 (1.2)	10 (10.1)	**< 0.001**

CRP, C-reactive protein; WBC, white blood cell; HbA1c, glycated hemoglobin; MCA, middle cerebral artery; ASPECTS, Alberta Stroke Program Early CT Score; CBS, clot burden score; ICA, internal carotid artery; ICH, intracranial hemorrhage. Bold *P*-values indicate significance (*P* < 0.05).

### 3.4 Neuroimaging parameters

CTA-derived imaging parameters demonstrated striking disparities between the two outcome groups. Large infarction involving more than one-third of the MCA territory (corresponding to ASPECTS ≤ 7) was observed in 54 patients (54.5%) in the unfavorable outcome group compared with only 32 (18.9%) in the favorable outcome group (*P* < 0.001). The median ASPECTS was significantly lower in the unfavorable group [6 (IQR 5–8) vs. 9 (IQR 7–10); *P* < 0.001]. Regarding the collateral circulation, poor Tan scores (grades 0–1) were observed in 50 patients (50.5%) with unfavorable outcomes versus only 30 (17.7%) in the favorable group (*P* < 0.001). The median CBS was markedly lower in the unfavorable group, indicating greater thrombus burden [5 (IQR 3–7) vs. 8 (IQR 6–9); *P* < 0.001]. Proximal large vessel occlusion (ICA or M1) was present in 56.6% of patients with unfavorable outcomes compared with 30.8% in the favorable group (*P* < 0.001). Hemorrhagic transformation occurred in 34 patients (12.7%), with symptomatic intracranial hemorrhage observed in 12 (4.5%), both rates being significantly higher among patients with unfavorable outcomes (Table 2).

### 3.5 Univariate and multivariable logistic regression analysis

In univariate logistic regression analysis, 22 variables achieved statistical significance at the *P* < 0.10 threshold and were entered into the multivariable model (Supplementary Table 3). Following backward stepwise elimination, multivariable logistic regression identified seven independent predictors of unfavorable 3-month functional outcome: early neurological deterioration (adjusted OR 3.45, 95% CI 2.01–5.92; *P* < 0.001), large infarction exceeding one-third of the MCA territory (adjusted OR 2.78, 95% CI 1.58–4.89; *P* < 0.001), baseline NIHSS score (adjusted OR 1.14 per point, 95% CI 1.07–1.22; *P* < 0.001), poor Tan collateral score (adjusted OR 2.31, 95% CI 1.34–3.98; *P* = 0.003), low clot burden score (adjusted OR 2.15, 95% CI 1.28–3.61; *P* = 0.004), elevated D-dimer (adjusted OR 2.19, 95% CI 1.29–3.72; *P* = 0.004), and elevated CRP (adjusted OR 1.87, 95% CI 1.11–3.15; *P* = 0.019) (Table 3, with the corresponding forest plot of adjusted odds ratios provided in Supplementary Figure 7). The full model specification, comprising the regression intercept (β_0_ = –3.842), β coefficients, standard errors, Wald χ^2^ statistics, and shrinkage-adjusted coefficients, is provided in Supplementary Table 4 in accordance with TRIPOD reporting standards (Collins et al., 2015) to enable external recalibration and validation. Sensitivity analysis based on multiple imputation by chained equations confirmed close concordance with the complete-case estimates, with maximum deviation in adjusted ORs of < 8% across the seven retained predictors. Collinearity diagnostics confirmed that all variance inflation factors were below 2.0, indicating no problematic multicollinearity in the final model (Supplementary Table 6).

**TABLE 3:** Independent predictors of unfavorable 3-month outcome: multivariable logistic regression.

Predictor	Adjusted OR	95% CI	*P*-value
Early neurological deterioration	3.45	2.01–5.92	**< 0.001**
Large infarction ( > 1/3 MCA territory, ASPECTS ≤ 7)	2.78	1.58–4.89	**< 0.001**
NIHSS score (per point increase)	1.14	1.07–1.22	**< 0.001**
Poor Tan collateral score (grade 0–1)	2.31	1.34–3.98	**0.003**
Low clot burden score (per point decrease)	2.15	1.28–3.61	**0.004**
Elevated D-dimer	2.19	1.29–3.72	**0.004**
Elevated C-reactive protein	1.87	1.11–3.15	**0.019**

OR, odds ratio; CI, confidence interval; NIHSS, National Institutes of Health Stroke Scale; MCA, middle cerebral artery. Apparent model AUC = 0.847 (95% CI 0.801–0.893); optimism-corrected AUC = 0.831 (95% CI 0.785–0.877) on 1,000-replicate bootstrap internal validation; calibration slope = 0.948; intercept = 0.012; shrinkage factor = 0.948; Hosmer–Lemeshow *P* = 0.394. Model adjusted for age, sex, hypertension, diabetes mellitus, atrial fibrillation, blood glucose, onset-to-needle time, and all variables with *P* < 0.10 in univariate analysis. Full model specification including β*0* and shrinkage-adjusted coefficients is provided in Supplementary Table 4 in accordance with TRIPOD reporting standards. Bold values indicate statistically significant independent predictors (*P* < 0.05).

### 3.6 Model performance, internal validation, and clinical utility

**FIGURE 2:**
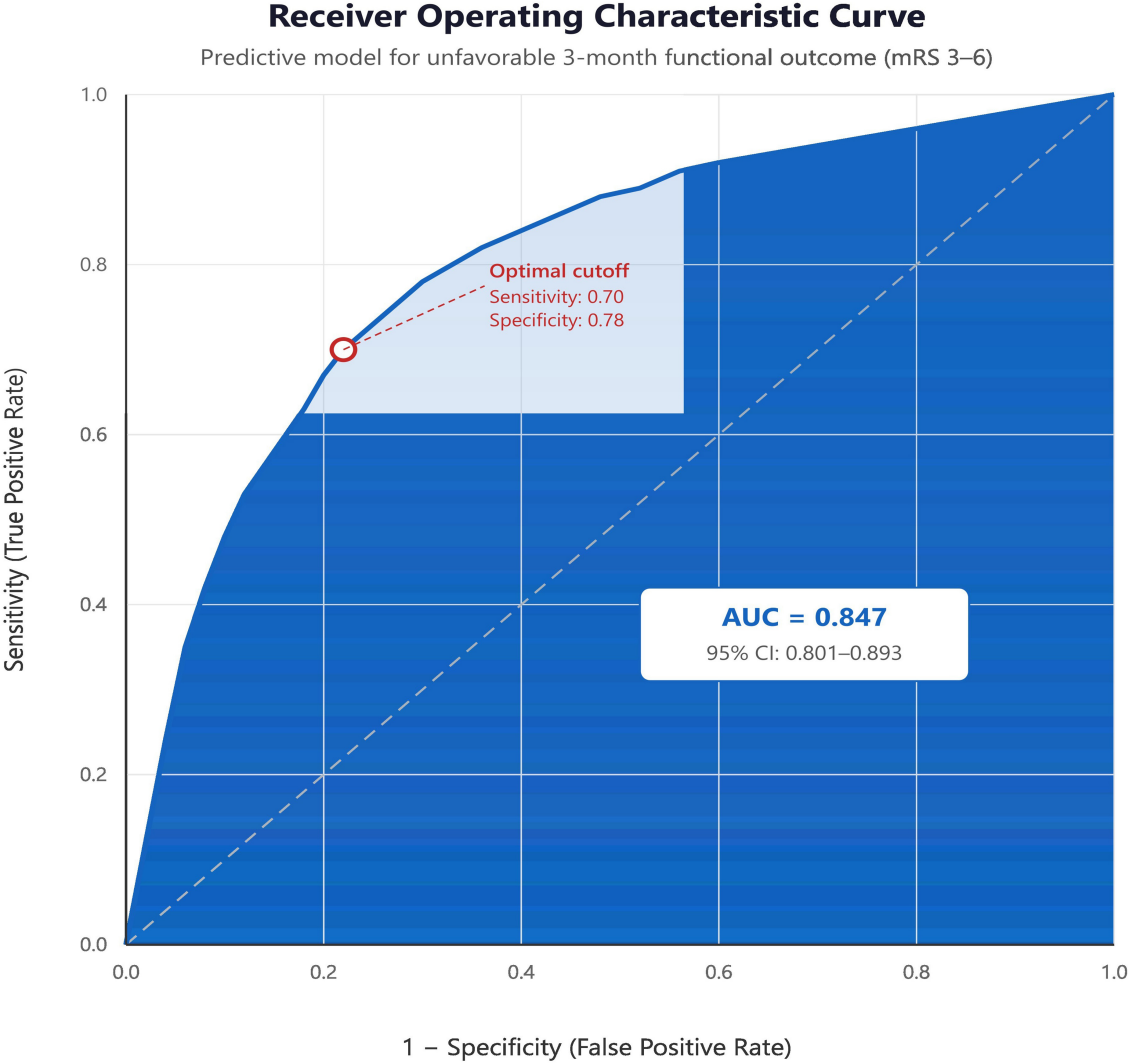
Receiver operating characteristic (ROC) curve for the composite seven-predictor multivariable logistic regression model predicting unfavorable 3-month functional outcome. The model achieved an area under the curve (AUC) of 0.847 (95% CI 0.801–0.893). The optimal cutoff point, identified by the maximal Youden index, yielded a sensitivity of 70.7% and specificity of 78.1%. The shaded area represents the 95% confidence region. The diagonal dashed line represents the line of no discrimination.

The composite seven-predictor model demonstrated excellent discriminative performance, with an apparent AUC of 0.847 (95% CI 0.801–0.893) for predicting unfavorable 3-month outcome (Figure 2). At the optimal cutoff derived from the maximal Youden index, the model achieved a sensitivity of 70.7%, specificity of 78.1%, positive predictive value of 65.4%, and negative predictive value of 82.0%. The Hosmer–Lemeshow goodness-of-fit test yielded a χ^2^ of 8.42 (*P* = 0.394), indicating satisfactory model calibration, a finding corroborated by the calibration plot (Supplementary Figure 3). The Nagelkerke *R*^2^ was 0.412, and the Brier score was 0.168, both indicating robust predictive accuracy (Supplementary Table 4). On 1,000-replicate bootstrap internal validation, the optimism-corrected AUC was 0.831 (95% CI 0.785–0.877), with a calibration slope of 0.948 and an intercept of 0.012, indicating only modest optimism and confirming the robustness of the apparent estimates; the corresponding linear shrinkage factor of 0.948 was applied to derive the shrunken regression coefficients reported in Supplementary Table 4. Decision curve analysis (Supplementary Figure 5) demonstrated that the model conferred a positive net clinical benefit across threshold probabilities ranging from approximately 0.15–0.75, exceeding the strategies of treating all or treating none of the patients within this clinically pertinent range. To facilitate bedside risk estimation, a nomogram was constructed from the final model coefficients (Supplementary Figure 6), in which each predictor is mapped onto an upper points axis whose summed score corresponds to the predicted probability of unfavorable 3-month outcome on a lower probability axis.

### 3.7 Head-to-head comparison with previously published prediction models

To position the discriminative performance of the present model within the existing prognostic literature, we recalibrated three previously published reference models within our cohort and compared their performance using the DeLong test for paired ROC curves (DeLong et al., 1988). The model of Hu et al. (2022) achieved an AUC of 0.756 (95% CI 0.699–0.813), the model of Ping et al. (2022) achieved an AUC of 0.781 (95% CI 0.724–0.838), and the model of Lv et al. (2020) achieved an AUC of 0.802 (95% CI 0.748–0.856), all of which were inferior to the apparent AUC of 0.847 obtained for our composite model. Pairwise DeLong testing demonstrated statistically significant differences in favor of the present model against the Hu (*P* = 0.014) and Ping (*P* = 0.038) models, and a near-significant trend against the Lv model (*P* = 0.072). Detailed discrimination, calibration, and DeLong *P*-values for each pairwise comparison are presented in Supplementary Table 7.

### 3.8 Subgroup and interaction analyses

Pre-specified subgroup analyses demonstrated that the seven-predictor model was consistently associated with unfavorable outcome across all examined strata (Supplementary Figure 4 and Supplementary Table 5). Significant interactions were observed for baseline stroke severity, with the magnitude of the predictive effect being substantially greater in patients with NIHSS > 10 (OR 4.18, 95% CI 2.24–7.80) compared with those with NIHSS ≤ 10 (OR 1.92, 95% CI 1.08–3.41; P for interaction = 0.028). A significant interaction was also detected for collateral status, with the predictive association being more pronounced in patients with poor collateral circulation (OR 4.42, 95% CI 2.18–8.96) than in those with good collaterals (OR 1.86, 95% CI 1.08–3.20; P for interaction = 0.015). No significant interactions were observed across strata defined by age, sex, onset-to-needle time, hypertension, diabetes mellitus, or atrial fibrillation.

## 4 Discussion

In this prospective cohort of 268 anterior circulation AIS patients treated with intravenous alteplase, we identified seven independent predictors of unfavorable 3-month functional outcome—early neurological deterioration, large infarction, baseline NIHSS score, poor collateral circulation, low clot burden score, elevated D-dimer, and elevated CRP—that together constituted a multimodal predictive model with an apparent AUC of 0.847 and an optimism-corrected AUC of 0.831 on 1,000-replicate bootstrap internal validation. These findings provide supportive evidence that the integration of clinical severity markers, inflammatory and coagulation biomarkers, and CTA-derived vascular imaging metrics yields a comprehensive prognostic framework that performs at least comparably to, and on head-to-head recalibration superior to, models relying on a single domain of assessment. The unfavorable outcome rate of 36.9% in our cohort is concordant with the existing literature, which has reported rates of 16–50% depending on the definitions employed and the populations studied (Fekete et al., 2023; Xu et al., 2025; Zhai et al., 2023).

An analytical decision deserving explicit comment relates to the deliberate inclusion of early neurological deterioration as a binary post-treatment predictor while electing not to enter the absolute 24-h NIHSS as a separate continuous covariate in the same model. Although the 24-h NIHSS has been shown in prior studies to be a strong predictor of outcome in both intravenous-only and endovascular populations, the construct is conceptually subsumed within the END definition (NIHSS increase ≥ 4 points), and in preliminary multivariable modeling the 24-h NIHSS exhibited a variance inflation factor of 4.18, approaching our pre-specified collinearity threshold and destabilizing the regression coefficients of the remaining predictors. We therefore retained END as the parsimonious post-treatment indicator and acknowledge that future investigations modeling the temporal trajectory of NIHSS through time-varying covariate or joint modeling approaches may further refine prognostic accuracy.

The identification of early neurological deterioration as the strongest independent predictor (adjusted OR 3.45) is consistent with its well-established role as a harbinger of poor prognosis in thrombolysis-treated patients, likely reflecting failed recanalization, reocclusion, or secondary complications such as hemorrhagic transformation and cerebral edema (Guo et al., 2024; Seners et al., 2015). The baseline NIHSS score, which quantifies the severity of neurological impairment at the time of presentation, emerged as a significant predictor with an adjusted OR of 1.14 per point increase, corroborating the findings of multiple prior investigations, including a recent large-scale retrospective study by Xu et al. (2025) involving 3,805 thrombolysis-treated patients that confirmed advancing age and NIHSS as the dominant prognostic determinants (Xu et al., 2025). Similarly, Zhai et al. (2023) identified baseline NIHSS as an independent predictor of 1-year unfavorable outcomes in 222 thrombolysis-treated patients (Zhai et al., 2023).

The prognostic significance of elevated D-dimer levels (adjusted OR 2.19) in our study resonates with the emerging paradigm that coagulation activation and fibrinolytic resistance are critical modulators of post-thrombolysis outcome. Jin et al. (2022) demonstrated that post-thrombolytic D-dimer elevation was independently associated with both symptomatic intracranial hemorrhage and poor functional outcome, with follow-up D-dimer achieving an AUC of 0.73 for outcome prediction (Jin et al., 2022). Furthermore, Li et al. (2022) reported that low D-dimer at 48 h following thrombolysis was an independent predictor of favorable functional outcome, suggesting that D-dimer dynamics may serve as a real-time biomarker of thrombolytic efficacy and ongoing thrombotic processes (Li et al., 2022). Elevated D-dimer may reflect a prothrombotic milieu that impedes successful recanalization, promotes reocclusion, or indicates extensive upstream thrombotic activity that is inadequately addressed by pharmacological fibrinolysis alone. The association between D-dimer and thrombectomy outcomes has also been described, with Xie and Tang (2024) reporting that lower admission D-dimer predicted successful first-pass recanalization in mechanical thrombectomy (Xie and Tang, 2024). In a related study involving the MR CLEAN-NO IV trial, Barakzie et al. (2025) observed correlations between elevated D-dimer levels and unfavorable long-term functional outcomes as well as larger infarct sizes following thrombectomy (Barakzie et al., 2025).

The independent predictive value of elevated CRP (adjusted OR 1.87) underscores the pathogenic role of systemic inflammation in modulating stroke outcome. Post-ischemic neuroinflammation exacerbates blood-brain barrier disruption, augments cerebral edema, amplifies excitotoxic injury, and promotes hemorrhagic transformation, all of which attenuate the potential benefit of reperfusion therapy (VanGilder et al., 2014; Welsh et al., 2008). Li et al. (2023) provided convergent evidence by demonstrating that plasma levels of neutrophil serine proteinases, molecular effectors of the innate inflammatory cascade, were independent predictors of 3-month unfavorable outcome in AIS patients, including those treated with intravenous thrombolysis, with the addition of inflammatory biomarkers significantly improving reclassification metrics beyond traditional clinical predictors (Li et al., 2023). The mechanistic convergence of inflammatory and coagulation pathways further substantiates the value of integrating CRP and D-dimer alongside clinical and imaging variables, since post-ischemic activation of the innate immune system promotes endothelial dysfunction, microvascular thrombosis, and thromboinflammatory injury, processes that together help explain why CRP and D-dimer carry complementary prognostic information beyond conventional clinical scales.

The CTA-derived vascular parameters, namely poor Tan collateral score and low CBS, contributed substantively to the predictive model, with adjusted ORs of 2.31 and 2.15, respectively. These findings affirm the central importance of vascular anatomy in determining tissue fate following thrombolysis. The collateral circulation functions as an endogenous compensatory mechanism that sustains perfusion to the ischemic penumbra during arterial occlusion, thereby prolonging the viability of salvageable tissue and extending the effective therapeutic window (Menon et al., 2011; Tan et al., 2009). Patients with robust collateral networks are more likely to maintain penumbral perfusion until reperfusion is achieved, translating into smaller final infarct volumes and better functional outcomes. Conversely, the CBS serves as a quantitative surrogate for thrombus extent in the anterior circulation; a lower score signifies a larger clot burden, which has been consistently associated with reduced recanalization rates following intravenous thrombolysis and poorer functional outcomes (Puetz et al., 2008; Szegedi et al., 2021). Szegedi et al. (2021) confirmed that CBS is a significant independent predictor of both short- and long-term functional outcomes in AIS patients treated with intravenous thrombolysis (Szegedi et al., 2021). The Tan collateral score and CBS therefore capture hemodynamic and anatomical dimensions of stroke pathophysiology that are inadequately reflected by clinical severity scores alone, and their inclusion confers a distinctive advantage to the integrated model demonstrated in the present study.

Our finding of significant interactions between the predictive model and both baseline stroke severity (P for interaction = 0.028) and collateral status (P for interaction = 0.015) carries important clinical implications. The amplified predictive effect in patients with NIHSS > 10 and poor collaterals suggests that the prognostic relevance of the identified biomarkers is most pronounced among patients with the greatest burden of ischemic injury and the least hemodynamic reserve, precisely the subgroup in whom early risk stratification may exert the greatest influence on clinical decision-making, including the escalation to endovascular therapy or the initiation of intensive neuroprotective strategies.

Wang et al. (2024) recently developed and externally validated a nomogram incorporating baseline NIHSS, D-dimer, random blood glucose, blood urea nitrogen, and systolic blood pressure for predicting unfavorable outcomes following reperfusion therapy, achieving AUC values of 0.865 in the training set and 0.779 in external validation (Wang et al., 2024). To position our findings within this expanding prognostic landscape, we additionally recalibrated the prediction models of Hu et al. (2022), Ping et al. (2022), and Lv et al. (2020) within the present cohort, with our composite model demonstrating numerically and, for two of the three comparisons, statistically superior discrimination on the DeLong test (Supplementary Table 7). The clinical utility implications of these findings are reinforced by the decision curve analysis, in which the model conferred a positive net benefit across threshold probabilities of 0.15–0.75, a range that translates operationally into clinical decisions such as the consideration of intensified neurological monitoring, neurocritical care escalation, and the early discussion of additional therapeutic strategies for patients identified as high-risk. Because early neurological deterioration is ascertained during the first 24 h after thrombolysis, the present model is intended for application at the post-treatment 24-h clinical reassessment rather than at initial presentation, and the prognostic estimates it generates should be interpreted as conditional on the patient’s clinical trajectory through the first day after intravenous alteplase. Nonetheless, in the absence of external validation, claims of superior generalizability remain provisional, and we have therefore tempered our interpretive language accordingly. The complementary predictive contributions of inflammatory (CRP), coagulation (D-dimer), clinical (NIHSS, END), and imaging (Tan score, CBS, large infarction) parameters in our model argue strongly for the adoption of an integrated approach to prognostic assessment in the acute stroke setting.

This study possesses several methodological strengths, including its prospective design, the consecutive enrollment of patients, the comprehensive assessment of clinical, laboratory, and imaging parameters, the standardized 3-month outcome evaluation, and the rigorous multivariable analytical framework with collinearity diagnostics, calibration assessment, bootstrap internal validation with optimism correction and shrinkage, decision curve analysis, head-to-head benchmarking against established reference models, and pre-specified subgroup analyses. Nonetheless, several limitations warrant acknowledgment. First, the single-center design and the geographic concentration of the cohort within a single tertiary referral hospital may restrict the generalizability of our findings to populations with different demographic, ethnic, or clinical profiles, and external validation in independent multicenter cohorts is therefore essential. Second, the sample size of 268 patients, while adequate for the identification of seven independent predictors, may have limited the statistical power of certain subgroup analyses and precluded the detection of weaker but potentially meaningful associations. Third, laboratory parameters were measured at a single time point (admission), and serial assessments of D-dimer and CRP dynamics during the post-thrombolysis period may have provided additional prognostic information. Fourth, posterior circulation strokes, patients receiving tenecteplase, and patients receiving endovascular thrombectomy were excluded, thereby limiting the applicability of our findings to anterior circulation AIS treated with pharmacological alteplase alone and constraining their relevance to contemporary populations managed with combined or stand-alone endovascular therapy. Fifth, advanced imaging parameters such as quantitative ischemic core volume, final infarct volume, and angiographic recanalization status were not available, since CT perfusion and post-treatment angiographic follow-up were not part of the institutional acute stroke protocol during the enrollment period; the incorporation of these parameters in future investigations may further enhance discriminative performance. Sixth, although 3-month functional status was assessed by trained, blinded raters using validated structured instruments (smRSq for in-person evaluation and RFA-A for telephone interview) with excellent inter-rater agreement, the use of mixed assessment modalities cannot fully exclude residual measurement variability. Seventh, the use of a complete-case analytical approach, while supported by Little’s MCAR test and a multiple-imputation sensitivity analysis, leaves a residual potential for subtle selection bias from case exclusion. Eighth, internal validation, while methodologically rigorous, does not substitute for external validation in geographically and demographically distinct cohorts, and such validation through planned multicenter collaboration represents the indispensable next step before the present model can be recommended for routine clinical implementation.

## 5 Conclusion

In this prospective cohort of anterior circulation AIS patients treated with intravenous alteplase, early neurological deterioration, large infarction, baseline NIHSS score, poor collateral circulation, low clot burden score, elevated D-dimer, and elevated CRP were identified as seven independent predictors of unfavorable 3-month functional outcome. The composite multimodal predictive model demonstrated robust discrimination (apparent AUC 0.847; optimism-corrected AUC 0.831) and satisfactory calibration, with a positive net clinical benefit on decision curve analysis, supporting the integration of clinical, biochemical, and neuroimaging parameters for early prognostic stratification. These findings hold the potential to inform individualized risk assessment, facilitate targeted therapeutic interventions, and guide patient and family counseling in the acute phase of stroke care, but should be regarded as hypothesis-generating until corroborated through external validation in geographically and demographically distinct multicenter cohorts.

## Data Availability

The raw data supporting the conclusions of this article will be made available by the authors, without undue reservation.

## Supplementary material

Supplementary Figure 1 Bar chart depicting the distribution of individual modified Rankin Scale (mRS) scores at 3-month follow-up among the 268 study participants. Favorable outcome (mRS 0–2) was achieved by 169 patients (63.1%), comprising mRS 0 (*n* = 25, 9.3%), mRS 1 (*n* = 65, 24.3%), and mRS 2 (*n* = 79, 29.5%). Unfavorable outcome (mRS 3–6) was observed in 99 patients (36.9%), comprising mRS 3 (*n* = 42, 15.7%), mRS 4 (*n* = 30, 11.2%), mRS 5 (*n* = 15, 5.6%), and mRS 6 (*n* = 12, 4.5%).

Supplementary Figure 2 Box-and-whisker plots comparing the distributions of D-dimer (mg/L), C-reactive protein (mg/L), and baseline NIHSS score between the favorable (mRS 0–2) and unfavorable (mRS 3–6) outcome groups. These three variables were selected as illustrative biomarkers spanning three complementary pathophysiological domains, namely coagulation activation, systemic inflammation, and clinical severity, in order to provide a focused visual summary of the most clinically actionable continuous predictors; complete laboratory and imaging profiles for all parameters are tabulated in Tables 1, 2 and Supplementary Tables 1, 2. Median values (horizontal lines within boxes), interquartile ranges (box boundaries), and whiskers (extending to 1.5 × IQR) are displayed. All three parameters demonstrated significantly higher values in the unfavorable outcome group (*P* < 0.001 for all comparisons). Asterisks denote statistical significance: ****P* < 0.001.

Supplementary Figure 3 Calibration plot of the seven-predictor multivariable logistic regression model. Predicted probabilities of unfavorable outcome (*x*-axis) are plotted against observed probabilities (*y*-axis) across decile groups. The dashed diagonal line represents perfect calibration. Close approximation of the observed data points to the diagonal indicates satisfactory model calibration, confirmed by the Hosmer–Lemeshow goodness-of-fit test (χ^2^ = 8.42; *P* = 0.394).

Supplementary Figure 4 Forest plot of subgroup analyses depicting odds ratios (with 95% confidence intervals) for unfavorable 3-month functional outcome across pre-specified subgroups stratified by age, sex, NIHSS severity, onset-to-needle time, hypertension, diabetes mellitus, atrial fibrillation, and collateral status. *P*-values for interaction are displayed alongside each pair of subgroup estimates. Significant interactions were observed for baseline NIHSS (*P* = 0.028) and collateral status (*P* = 0.015), indicating differential model performance across these strata.

Supplementary Figure 5 Decision curve analysis displaying the standardized net clinical benefit of the seven-predictor composite model (solid curve), the treat-all strategy (gray curve), and the treat-none strategy (horizontal dashed line) across the full range of threshold probabilities. The composite model conferred a positive net benefit superior to both reference strategies across threshold probabilities of approximately 0.15–0.75, a range corresponding to clinically pertinent decision contexts including the consideration of intensified neurological monitoring, neurocritical care escalation, and the early discussion of additional therapeutic strategies for patients identified as high-risk.

Supplementary Figure 6 Bedside nomogram derived from the final seven-predictor logistic regression model. Each predictor (early neurological deterioration, large infarction, baseline NIHSS, Tan collateral score, clot burden score, D-dimer, and C-reactive protein) is mapped onto an upper points axis. The summed total points score corresponds to the predicted probability of unfavorable 3-month functional outcome (mRS 3–6) on the lower probability axis, providing a transparent and operational framework for individualized risk estimation in the acute stroke setting.

Supplementary Figure 7 Forest plot illustrating the adjusted odds ratios (with 95% confidence intervals) derived from multivariable logistic regression analysis for the seven independent predictors of unfavorable 3-month functional outcome (mRS 3–6). The vertical dashed line at OR = 1 represents the null value. Diamond size is proportional to the magnitude of the odds ratio. Early neurological deterioration emerged as the strongest predictor (adjusted OR 3.45, 95% CI 2.01–5.92; *P* < 0.001), followed by large infarction (adjusted OR 2.78), poor Tan collateral score (adjusted OR 2.31), elevated D-dimer (adjusted OR 2.19), low clot burden score (adjusted OR 2.15), elevated CRP (adjusted OR 1.87), and baseline NIHSS (adjusted OR 1.14 per point).
