# Supplementary figures and images for: Predictors of unfavorable 3-month functional outcome following intravenous thrombolysis with alteplase in anterior circulation acute ischemic stroke: a prospective cohort study
Source: Front Neurosci. 2026 Jul 8;20:1818367. doi: 10.3389/fnins.2026.1818367 (PMC13388263; doi:10.3389/fnins.2026.1818367)

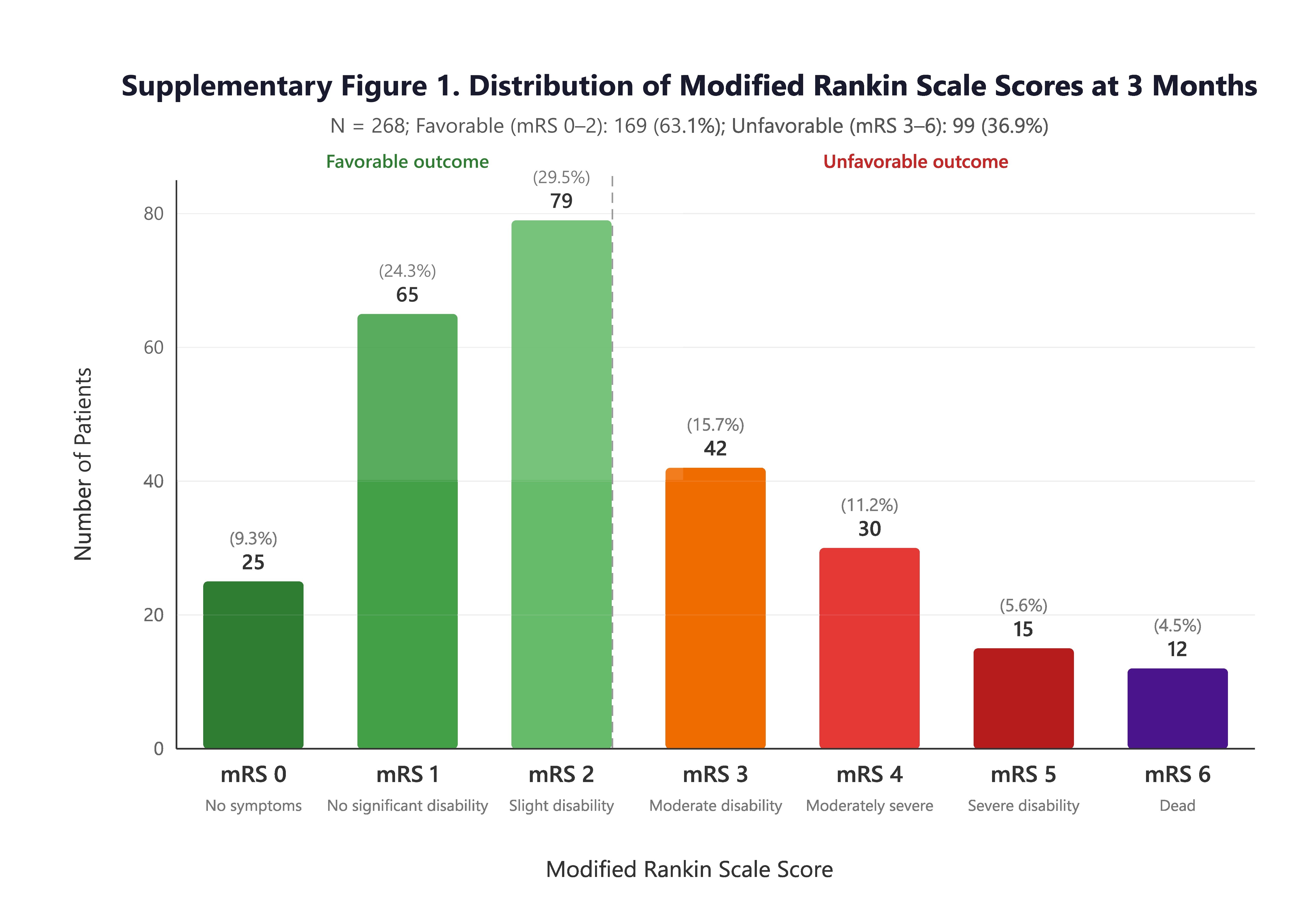

Supplement: Supplementary Figure 1 — Bar chart depicting the distribution of individual modified Rankin Scale (mRS) scores at 3-month follow-up among the 268 study participants. Favorable outcome (mRS 0–2) was achieved by 169 patients (63.1%), comprising mRS 0 (n = 25, 9.3%), mRS 1 (n = 65, 24.3%), and mRS 2 (n = 79, 29.5%). Unfavorable outcome (mRS 3–6) was observed in 99 patients (36.9%), comprising mRS 3 (n = 42, 15.7%), mRS 4 (n = 30, 11.2%), mRS 5 (n = 15, 5.6%), and mRS 6 (n = 12, 4.5%). [file Image_1.jpeg]

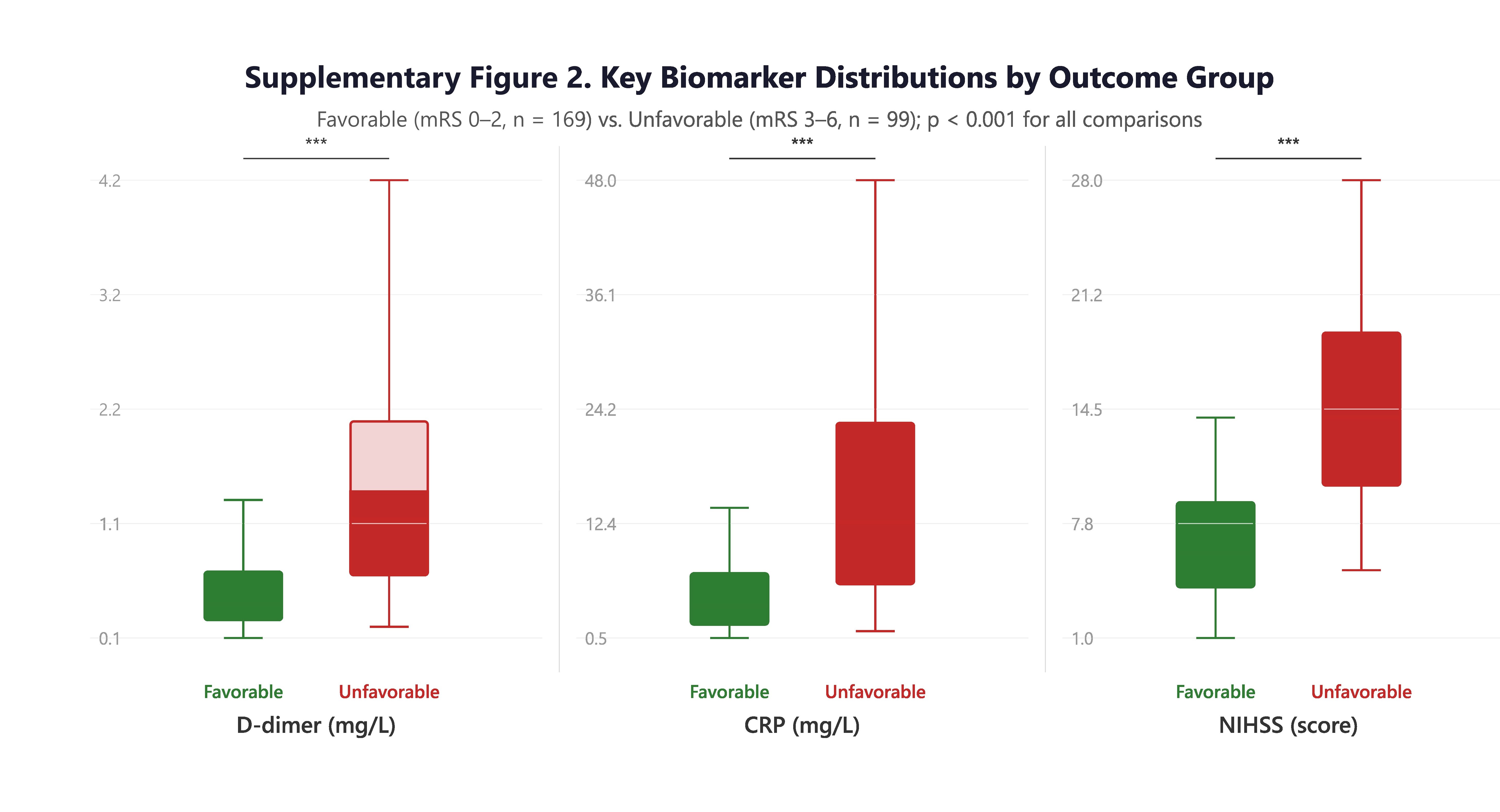

Supplement: Supplementary Figure 2 — Box-and-whisker plots comparing the distributions of D-dimer (mg/L), C-reactive protein (mg/L), and baseline NIHSS score between the favorable (mRS 0–2) and unfavorable (mRS 3–6) outcome groups. These three variables were selected as illustrative biomarkers spanning three complementary pathophysiological domains, namely coagulation activation, systemic inflammation, and clinical severity, in order to provide a focused visual summary of the most clinically actionable continuous predictors; complete laboratory and imaging profiles for all parameters are tabulated in Tables 1, 2 and Supplementary Tables 1, 2. Median values (horizontal lines within boxes), interquartile ranges (box boundaries), and whiskers (extending to 1.5 × IQR) are displayed. All three parameters demonstrated significantly higher values in the unfavorable outcome group (P < 0.001 for all comparisons). Asterisks denote statistical significance: ***P < 0.001. [file Image_2.jpeg]

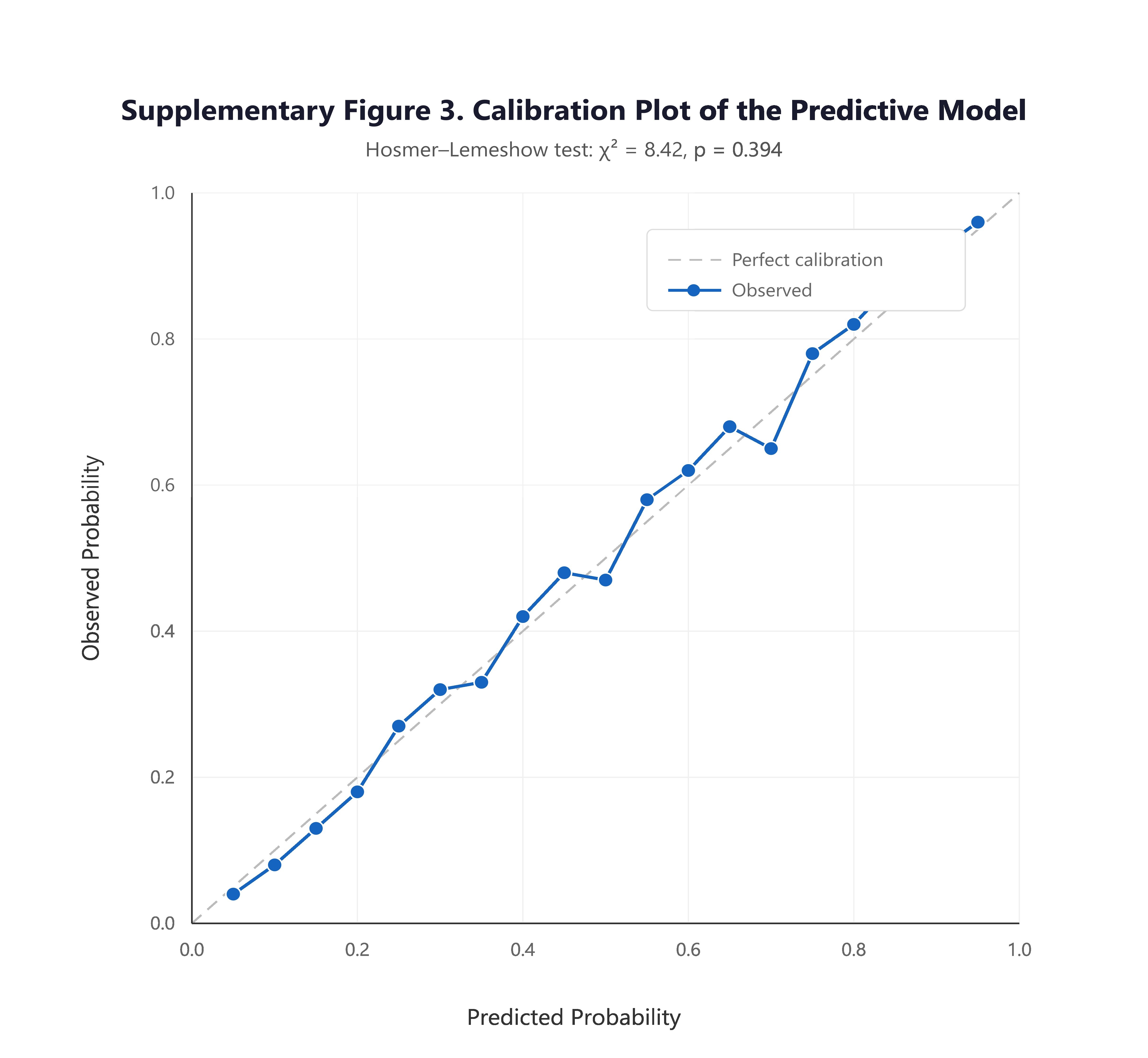

Supplement: Supplementary Figure 3 — Calibration plot of the seven-predictor multivariable logistic regression model. Predicted probabilities of unfavorable outcome (x-axis) are plotted against observed probabilities (y-axis) across decile groups. The dashed diagonal line represents perfect calibration. Close approximation of the observed data points to the diagonal indicates satisfactory model calibration, confirmed by the Hosmer–Lemeshow goodness-of-fit test (χ2 = 8.42; P = 0.394). [file Image_3.jpeg]

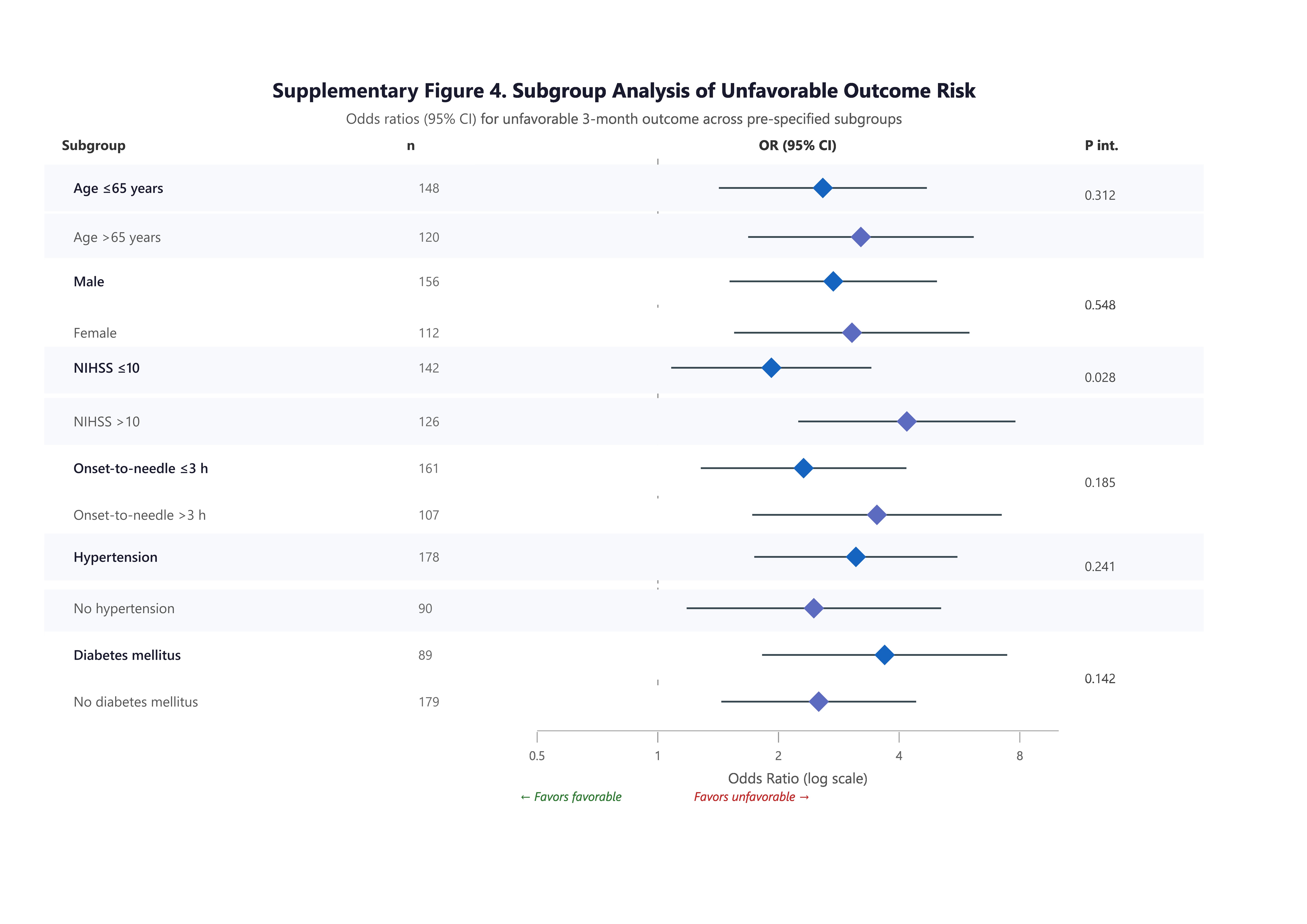

Supplement: Supplementary Figure 4 — Forest plot of subgroup analyses depicting odds ratios (with 95% confidence intervals) for unfavorable 3-month functional outcome across pre-specified subgroups stratified by age, sex, NIHSS severity, onset-to-needle time, hypertension, diabetes mellitus, atrial fibrillation, and collateral status. P-values for interaction are displayed alongside each pair of subgroup estimates. Significant interactions were observed for baseline NIHSS (P = 0.028) and collateral status (P = 0.015), indicating differential model performance across these strata. [file Image_4.jpeg]

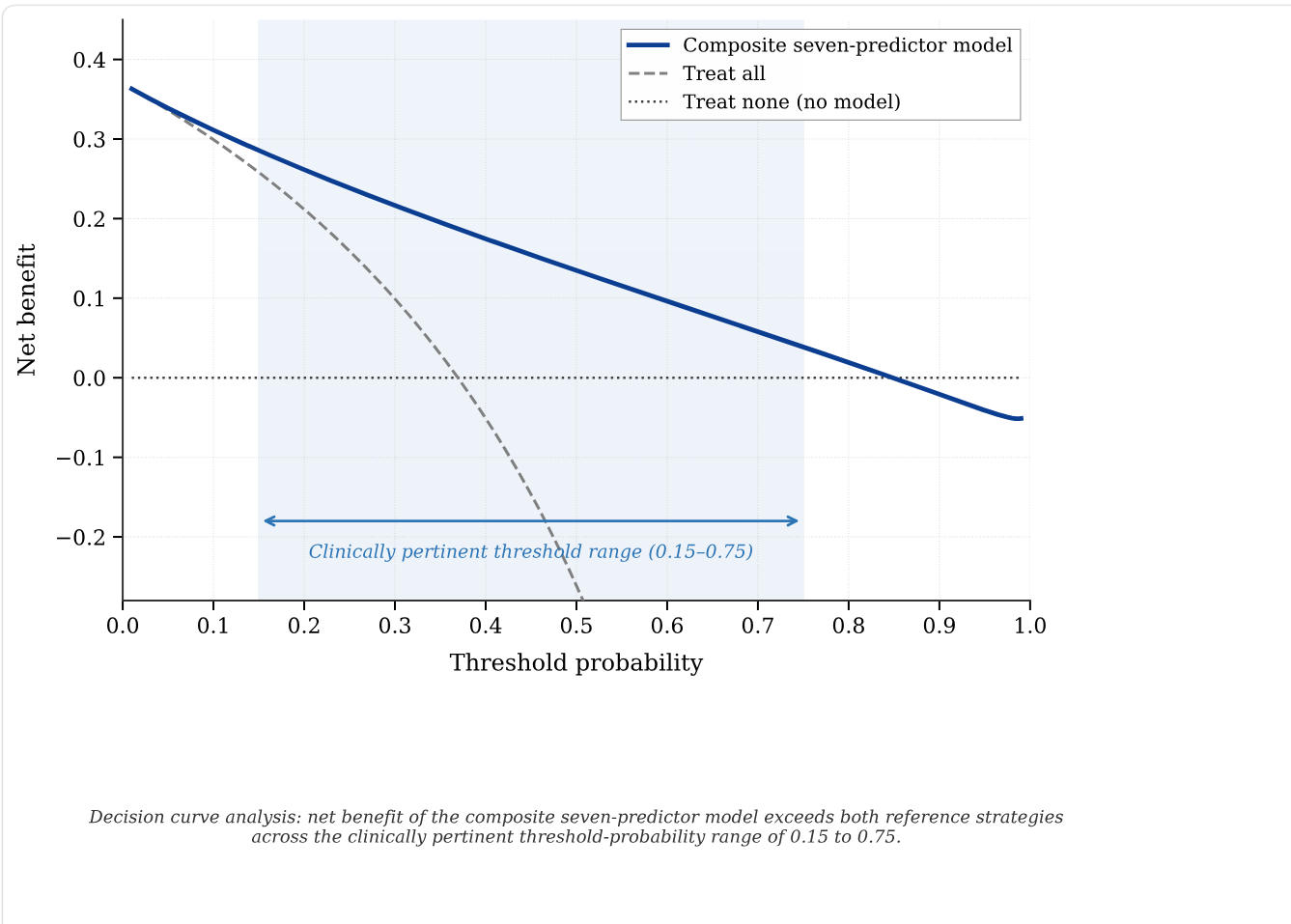

Supplement: Supplementary Figure 5 — Decision curve analysis displaying the standardized net clinical benefit of the seven-predictor composite model (solid curve), the treat-all strategy (gray curve), and the treat-none strategy (horizontal dashed line) across the full range of threshold probabilities. The composite model conferred a positive net benefit superior to both reference strategies across threshold probabilities of approximately 0.15–0.75, a range corresponding to clinically pertinent decision contexts including the consideration of intensified neurological monitoring, neurocritical care escalation, and the early discussion of additional therapeutic strategies for patients identified as high-risk. [file Image_5.jpeg]

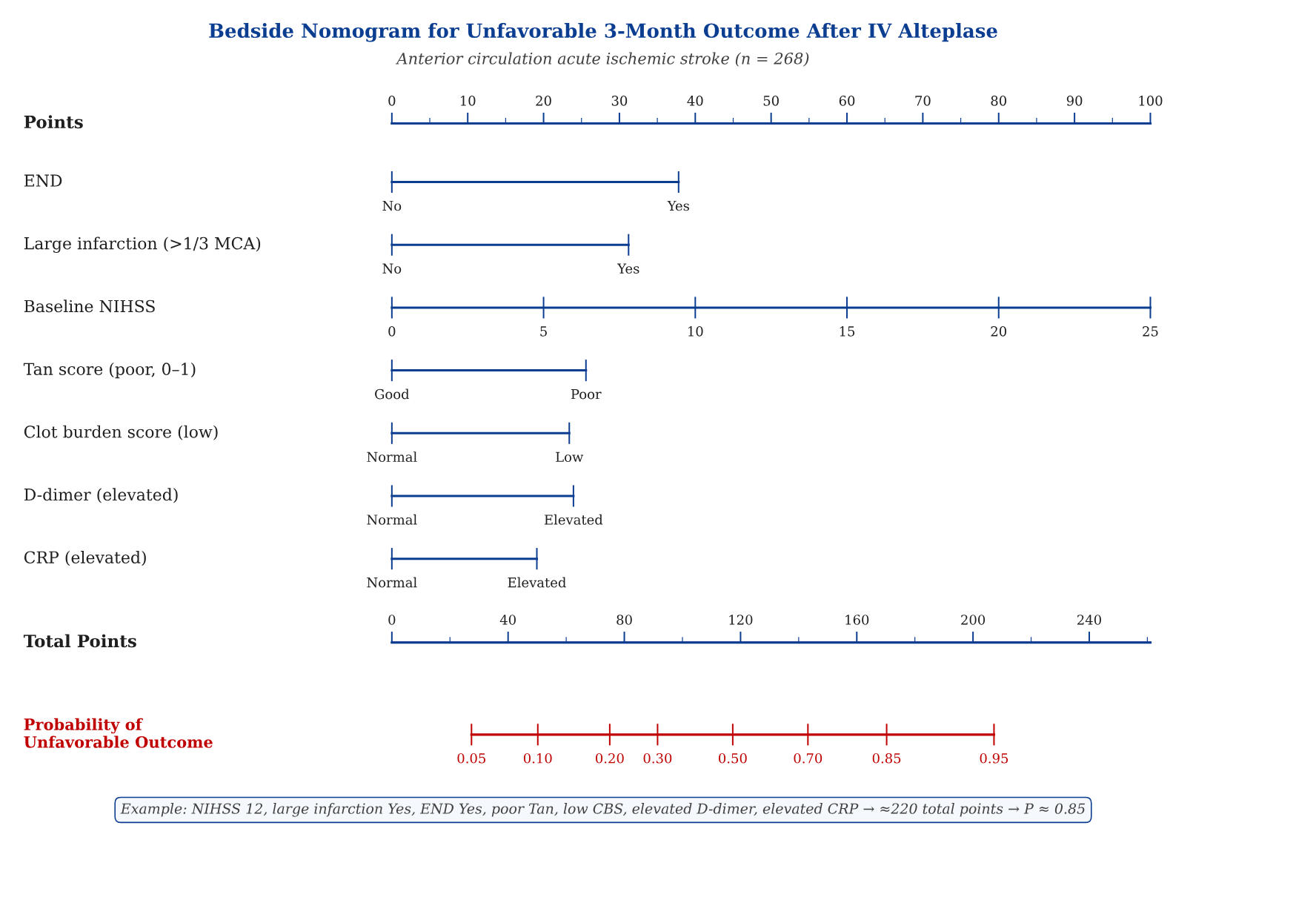

Supplement: Supplementary Figure 6 — Bedside nomogram derived from the final seven-predictor logistic regression model. Each predictor (early neurological deterioration, large infarction, baseline NIHSS, Tan collateral score, clot burden score, D-dimer, and C-reactive protein) is mapped onto an upper points axis. The summed total points score corresponds to the predicted probability of unfavorable 3-month functional outcome (mRS 3–6) on the lower probability axis, providing a transparent and operational framework for individualized risk estimation in the acute stroke setting. [file Image_6.jpeg]

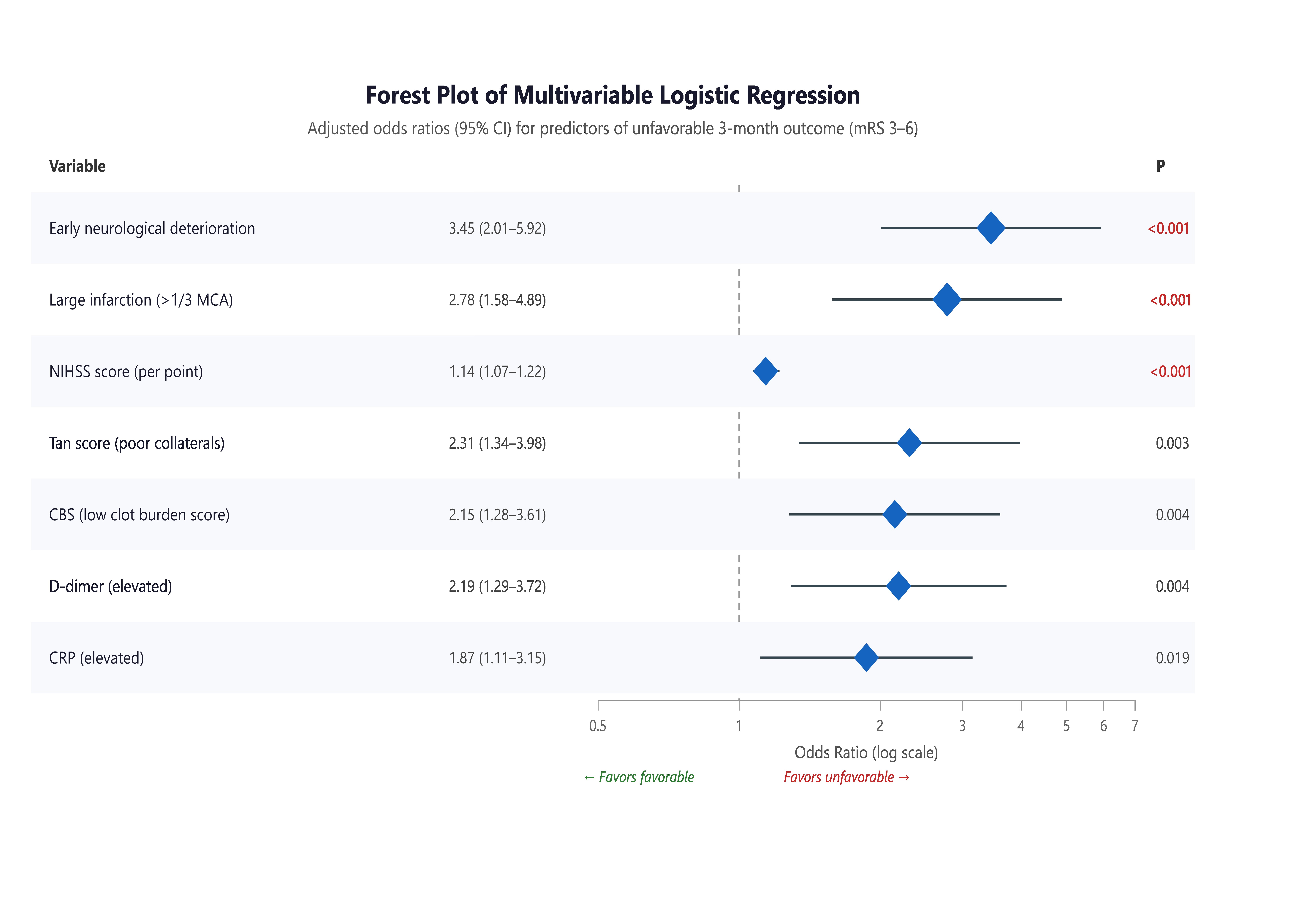

Supplement: Supplementary Figure 7 — Forest plot illustrating the adjusted odds ratios (with 95% confidence intervals) derived from multivariable logistic regression analysis for the seven independent predictors of unfavorable 3-month functional outcome (mRS 3–6). The vertical dashed line at OR = 1 represents the null value. Diamond size is proportional to the magnitude of the odds ratio. Early neurological deterioration emerged as the strongest predictor (adjusted OR 3.45, 95% CI 2.01–5.92; P < 0.001), followed by large infarction (adjusted OR 2.78), poor Tan collateral score (adjusted OR 2.31), elevated D-dimer (adjusted OR 2.19), low clot burden score (adjusted OR 2.15), elevated CRP (adjusted OR 1.87), and baseline NIHSS (adjusted OR 1.14 per point). [file Image_7.jpeg]
